# Intertypic reassortment of mammalian orthoreovirus identified in wastewater in Japan

**DOI:** 10.1038/s41598-021-92019-z

**Published:** 2021-06-15

**Authors:** Kouichi Kitamura, Hirotaka Takagi, Tomoichiro Oka, Michiyo Kataoka, Yo Ueki, Akie Sakagami

**Affiliations:** 1 Department of Virology II, National Institute of Infectious Diseases, 4-7-1 Gakuen, Musashi-murayama, Tokyo, 208-0011 Japan; 2 Management Department of Biosafety and Laboratory Animal, National Institute of Infectious Diseases, Tokyo, 208-0011 Japan; 3 Department of Pathology, National Institute of Infectious Diseases, Tokyo, 208-0011 Japan; 4 Miyagi Prefectural Institute of Public Health and Environment, Sendai, 983-0836 Japan

**Keywords:** Viral epidemiology, Water microbiology, Next-generation sequencing

## Abstract

Mammalian orthoreovirus (MRV), a non-enveloped virus with a ten-segmented double-stranded RNA genome, infects virtually all mammals, including humans. Human infection with MRV seems to be common in early childhood, but is rarely symptomatic. Despite the ubiquitous presence of MRV in mammals as well as in environmental waters, the molecular characterisation of the MRV genome remains to be fully elucidated. In this study, two novel strains, MRV-2 THK0325 and MRV-1 THK0617, were unintentionally isolated from wastewater in Japan via an environmental surveillance of enteric viruses. Homology and phylogenetic analysis demonstrated that all the segments of THK0325 were closely related to the MRV-2 Osaka strains, which were recently proposed to have existed for at least two decades in Japan. Most of the segments in THK0617 also showed a close relationship with the MRV-2 Osaka strains, but the M2, S1, and S3 segments belong to another MRV cluster. According to the S1 sequence, the determinant of serotype THK0617 was classified as MRV-1, and both the M2 and S3 segments were closely related to MRV-1 and -3 from the tree shrew in China. These results suggest that the MRV-2 Osaka-like strain spread widely throughout Japan, accompanied by intertypic reassortment occurring in East Asia.

## Introduction

Environmental surveillance using wastewater has been developed to survey enteric viruses such as poliovirus, enterovirus, norovirus, and sapovirus^1–6^. The use of PCR is the gold standard for the identification of nucleic acids from viruses isolated from wastewater samples via cell culture. However, PCR approaches require specific primer sets designed from previously identified viral sequences. Next generation sequencing (NGS) with random primer amplification has recently been used as an alternative approach for detecting both known and novel viral sequences. Mammalian orthoreovirus (MRV) is a non-enveloped virus belonging to the genus *Orthoreovirus* of the family *Reoviridae*, and is considered one of the most abundant viruses in environmental waters^7,8^. This virus infects all mammalian species, including humans. Human infection with MRV seems to be common in early childhood but is rarely symptomatic^9,10^. When symptoms do arise, the common manifestations are coryza, pharyngitis, cough^11^, and gastroenteritis^12,13^. Rare but severe symptoms include neurological diseases, such as meningitis^12–17^. MRV contains ten segments of a double-stranded RNA (dsRNA) genome, comprising three large (L1–L3), three medium (M1–M3), and four small (S1–S4) size segments^18^. MRVs are classified into four serotypes, MRV-1–4, based on the S1 segment encoding an attachment protein. The classic serotypes isolated from children in the 1950s are prototypes, type 1 Lang, type 2 Joes, type 3 Abney, and type 3 Dearing^19^. The fourth serotype (MRV-4 Ndelle) was isolated from a mouse^20^. Owing to the properties of segmented genomes, segment reassortment occurs between distinct MRV strains during co-infection in one cell, even in different serotypes^18^.

Despite the ubiquitous presence of MRV in mammals as well as in environmental waters^17,21^, sequence data of isolated strains registered in the database are limited, likely due to a lack of standard PCR primer sets to amplify the ten segments of the MRV genome. Only four MRV strains isolated in Japan have been registered in the NCBI GenBank database (https://www.ncbi.nlm.nih.gov/labs/virus/vssi, accessed November 19, 2020), all of which were type 2. Among these, three isolates were recently designated as MRV-2 Osaka strains, which were isolated from Osaka city (western Japan) in 1994, 2005, and 2014^13^. The present study investigated two novel MRV strains, identified by NGS analysis, in an attempt to survey enteric viruses from wastewater in the Tohoku region (north-eastern Japan). This is the first report on the molecular characterisation of a novel MRV isolate from wastewater in Japan.

## Results

**Figure 1:**
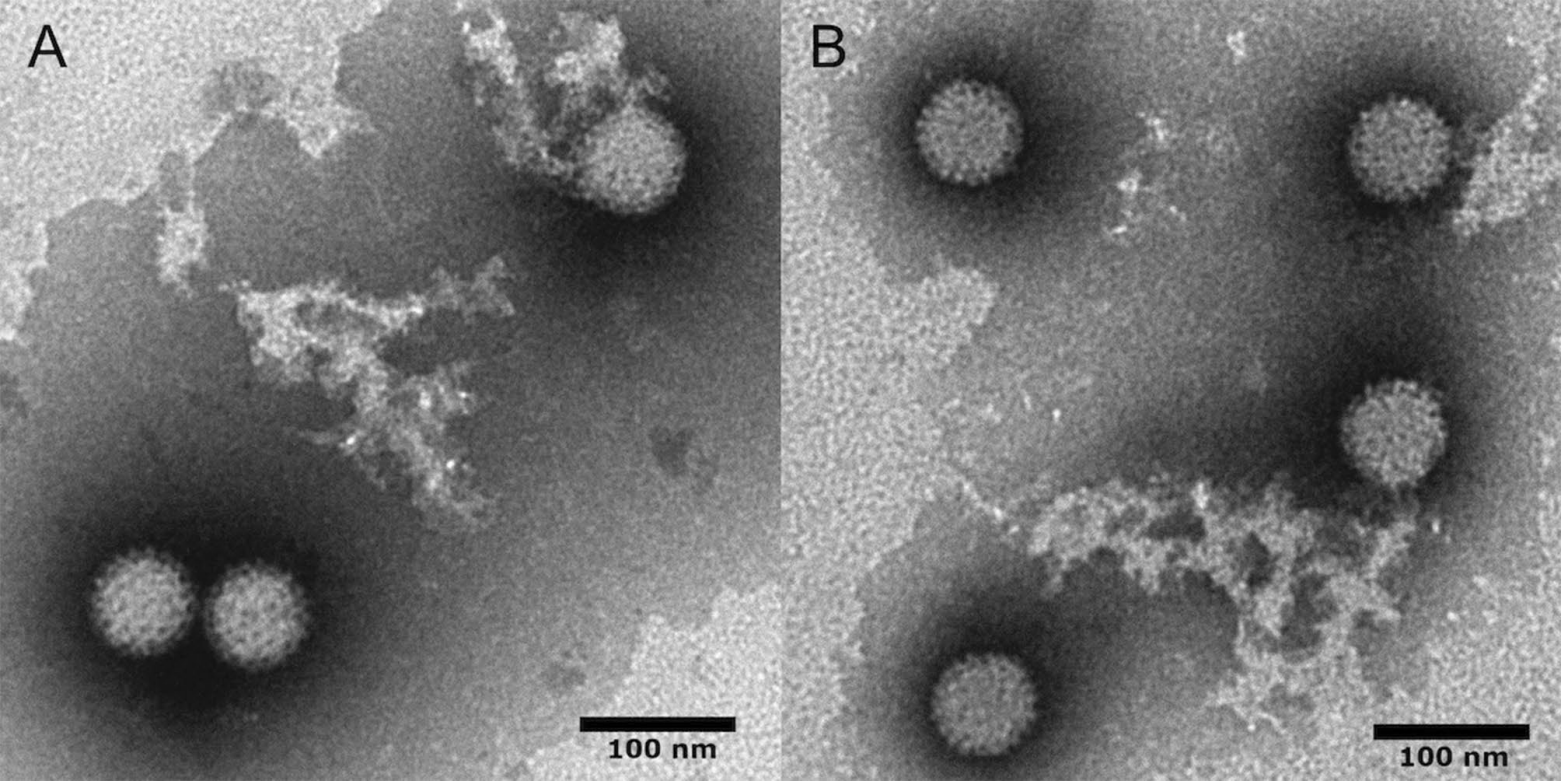
Identification of novel MRV isolates. Electron microscopic observation of novel MRV isolates, (**A**) THK0325 and (**B**) THK0617. Negatively stained virions showing icosahedral particles with a diameter of 60–70 nm. Bar = 100 nm.

To perform an environmental surveillance for enteric viruses, wastewater samples were collected weekly from the influent of a wastewater treatment plant (WWTP) located in the northeast region of Japan from March to June 2020, and used for virus isolation. Among the ten samples tested, THK0325 and THK0617 isolates were obtained from cell cultures with cytopathic effects (CPE). These isolates induced CPE in VeroE6, VeroE6/TMPRSS2, and HCT-8 cells, but not in OUMS-36T-2 cells. Transmission electron microscopy revealed a typical non-enveloped virion structure with a diameter of approximately 70 nm (Fig. 1). We then subjected these viral RNAs to NGS analysis using the MiSeq platform. Analysis with VirusTAP (virus genome-targeted assembly pipeline)^22^ covered the ten segments of the MRV genome, with complete or near-complete open reading frame (ORF) from both isolates.

**Figure 2:**
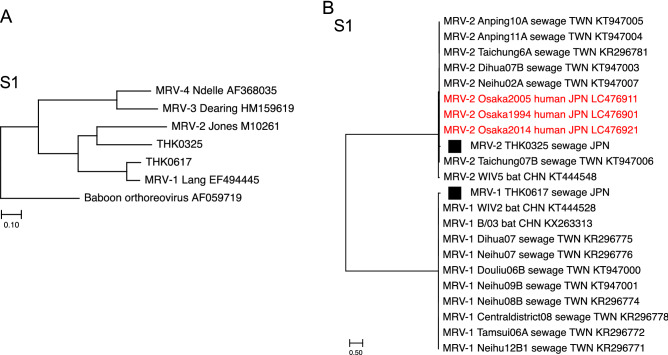
Phylogenetic analysis based on the nucleotide sequences of novel MRV isolates. Phylogenetic trees for the S1 segment were constructed through the maximum-likelihood method and 1000 bootstrap replicates using MEGA software. (**A**) Novel isolates and prototypes. A baboon orthoreovirus sequence was set as outgroup. (**B**) MRV sequences obtained from the GenBank database based on the best hits retrieval from the BLAST result. Each ID depicts the MRV genotype, name, host species, country, and accession number. Solid squares indicate the isolates in this study. Scale bars indicate genetic distances (nucleotide substitutions per site).

**Figure 3:**
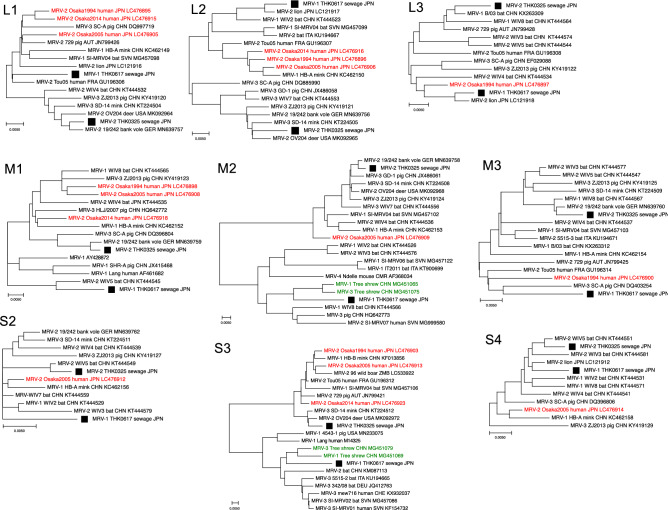
Phylogenetic analysis based on the nucleotide sequences of MRV isolates. Phylogenetic trees for segments aside from S1 were constructed through the maximum-likelihood method and 1000 bootstrap replicates using MEGA software. MRV sequences obtained from the GenBank database based on the best hits retrieval of the BLAST result. Each ID depicts the MRV genotype, name, host species, country, and accession number. Solid square: the isolates in this study. Red: MRV-2 Osaka strains. Green: MRV-1/TS/2011 and MRV-3/TS/2012. Scale bar: genetic distances (nucleotide substitutions per site).

**Figure 4:**
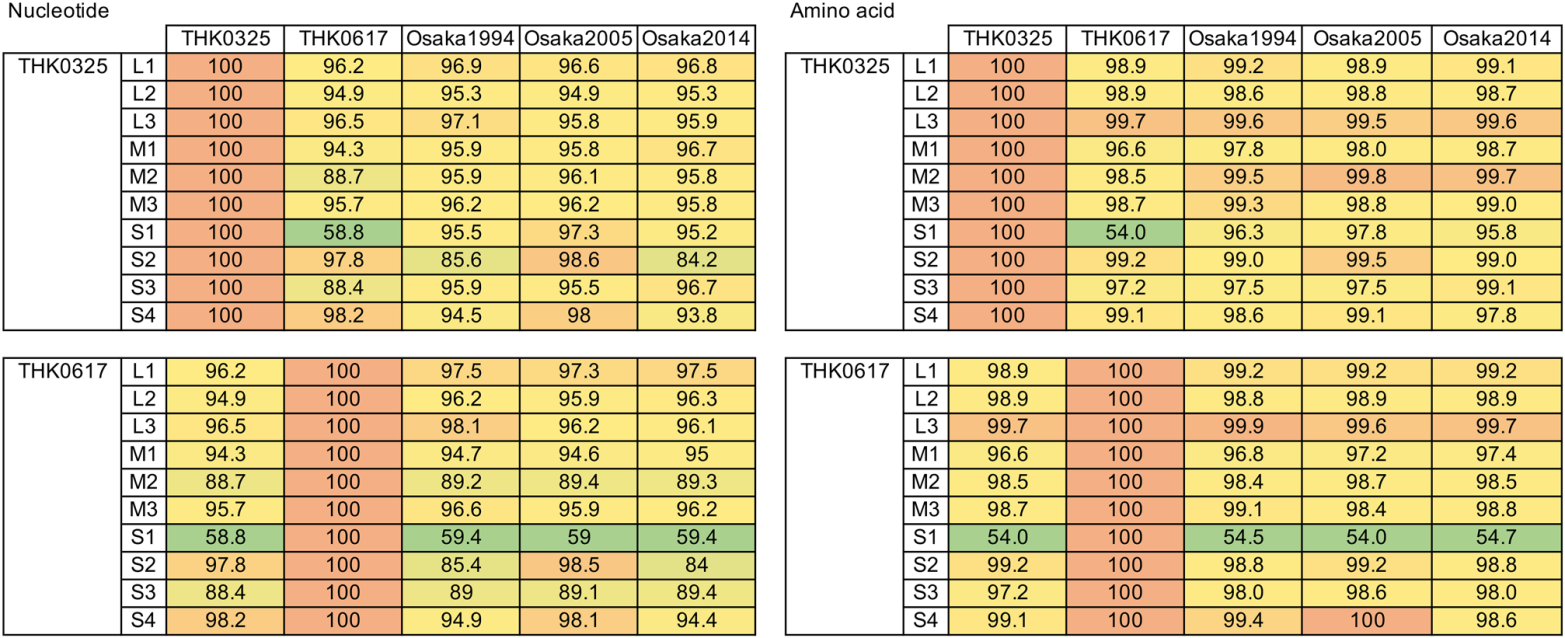
Pairwise sequence comparisons of MRV isolates. Nucleotide and amino acid identities between the novel isolates and MRV-2 Osaka strains previously isolated in Japan. Three-color scale formatting was applied to highlight the percentage values.

Phylogenetic analysis of S1 segments of both isolates with four prototypes showed that THK0325 was classified as MRV-2 and THK0617 as MRV-1 (Fig. 2A,B). To further confirm this, a search was carried out using a basic local alignment search tool (BLAST), with the S1 segments in the NCBI GenBank database. Analysis of S1 segments with other strains from the database showed that the THK0325 isolate belonged to the MRV-2 cluster containing Osaka and Taiwan strains^13,23^, and the THK0617 isolate was closely related to the Taiwan MRV-1 strains as well as WIV2 and B/03 bat strains in China^24,25^ (Fig. 2 and Supplementary Fig. 1). Phylogenetic analyses of THK0325 and THK0617 further demonstrated that the M2 and S3 segments of both isolates belonged to separate clusters (Fig. 3), and the segments of THK0325 were related to those of the MRV-2 Osaka strains, and the segments of THK0617 were closely related to strains from tree shrews in China^26^. Pairwise nucleotide identity comparison of all ten segments of the two isolates with the three Osaka strains is shown in Fig. 4. All ten segments of THK0325 and L1–L3, M1, M3, S2, and S4 of THK0617 showed high nucleotide identity with the Osaka strains, especially Osaka2005 (more than 94.9%). In addition to the S1 mentioned above, the M2 and S3 segments of THK0617 showed relatively low nucleotide identity (less than 90%) compared with the isolates in Japan, even though the amino acid identities were high (more than 96%). Taken together, our data indicate that the novel isolates are similar to the MRV-2 Osaka2005 strain, but M2, S1, and S3 segments of one isolate arose through multiple intertypic reassortment events.

## Discussion

In this study, two novel MRV strains were isolated from wastewater samples in the northeast region of Japan via environmental surveillance for enteric viruses. A previous report proposed that the MRV-2 Osaka strain had been circulating in Japan for more than two decades, although the viruses were only isolated from Osaka city^13^. The sample collection area in this study is located more than 500 km from Osaka city, and water bodies, including rivers and lakes, in both areas are independent. The analysis showed that the segments in the THK0325 isolate were closely related to the MRV-2 Osaka strains, especially the Osaka2005 isolate. In the other isolate THK0617, L1–L3, M1, M3, S2, and S4 segments also showed a close relationship to the Osaka2005 strain, but M2, S1, and S3 segments belonged to another cluster in the phylogenetic analysis (Figs. 2, 3). These results suggest that the MRV-2 Osaka-like strain spread widely throughout Japan, accompanied by segment reassortment.

**Figure 5:**
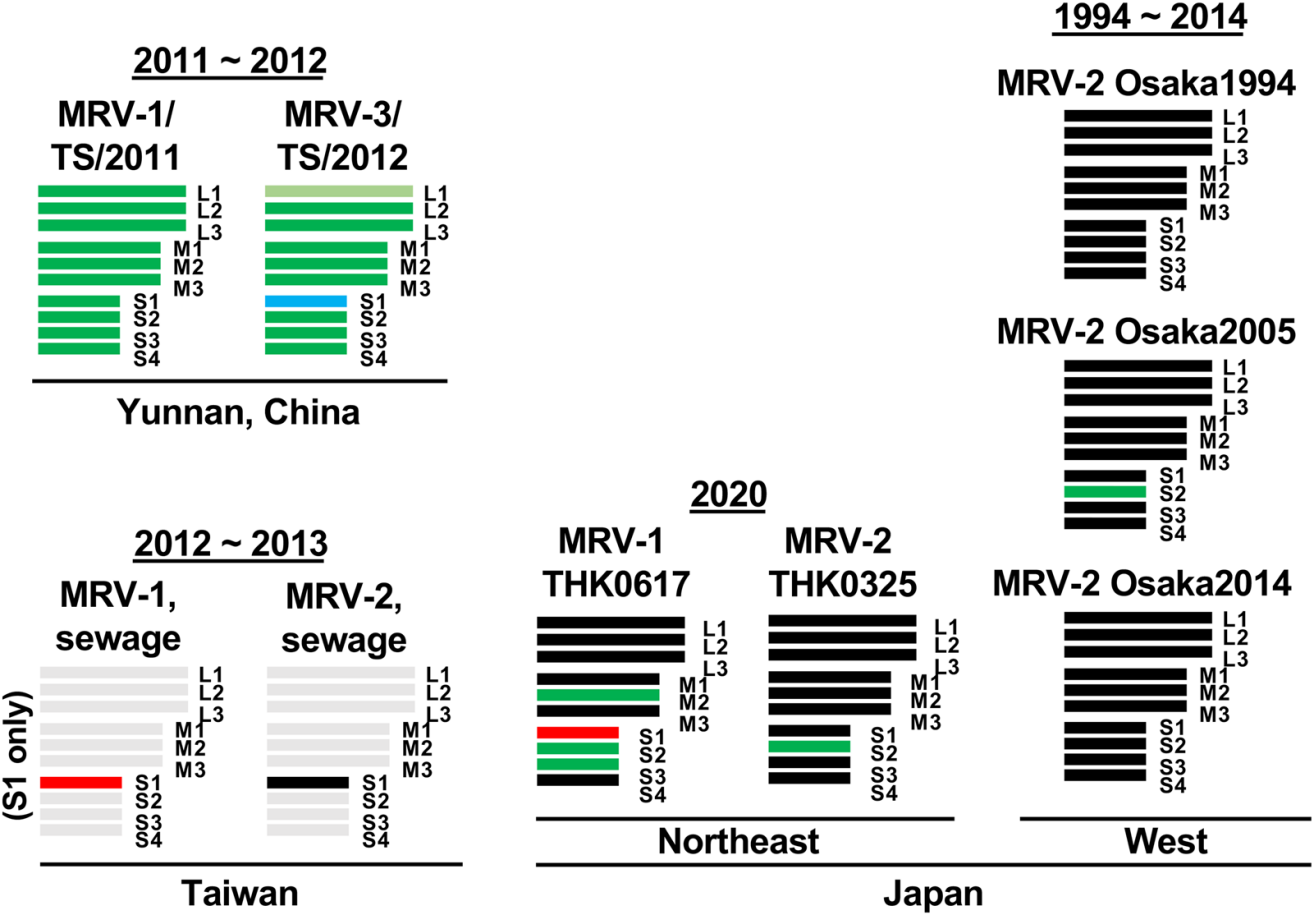
Schematic summary of the genomic compositions of novel MRV isolates and related strains. Based on the nucleotide identity and phylogenetic analysis, segments with close relation were distinguished by colour. Segments of THK0325 and THK0617 showing high nucleotide identity (more than 90% in Fig. 4) with Osaka1994 are in black. In Taiwan strains, S1 of MRV-1 and MRV-2 were closely related to THK0617 (red) and THK0325 (black), respectively (Fig. 2). S2 segments of Osaka2005 and THK0325, and M2, S2, and S3 segments of the THK0617 were related to MRV-1/TS/2011 and MRV-3/TS/2012 (green) (Fig. 3). The sole MRV-3 S1 segment is in light blue, and the unidentified segments of the Taiwan strain are in grey.

While the precise genetic flow cannot be determined, a possible scheme of the reassortment events is presented in Fig. 5. The analysis of S1 segments indicated that THK0617 was classified as MRV-1, suggesting that intertypic reassortment occurred between the Osaka MRV-2 strain and the unidentified MRV-1 strain. MRV-2 is considered the most prevalent serotype in Japan, and all of the molecularly characterised isolates were type 2. MRV-1 has not been identified even in a seroepidemiological survey since 2005 in Japan^13^. Identification of THK0617 as MRV-1 suggests that type 1 is still circulating in Japan and could exchange segments with the MRV-2 Osaka strain. The S1 segments of MRV-2 Osaka strains and MRV-2 Taiwan strains comprise a cluster with great diversity from other strains (less than 80% identity in nucleotide and amino acid levels) (Supplementary Fig. 1)^13^. S1 of THK0325 belongs to this cluster (Fig. 2B and black colour in Fig. 5). Interestingly, the S1 of THK0617 was also closely related to the Taiwan strain MRV-1 (Fig. 2B and red in Fig. 5). These results indicate a similar lineage between the novel isolates. The M2 and S3 segments of THK0617 were closely related to two isolates from the tree shrew in China, MRV-1/TS/2011 and MRV-3/TS/2012 (Fig. 3). Among the three Osaka strains, the S2 segment of Osaka2005 showed low nucleotide identity (less than 87%) compared with the other two Osaka isolates and belonged to a different cluster in which the tree shrew isolates belonged^13^ (Figs. 3, 4). Thus, the M2, S2, and S3 segments of the THK0617 isolate were related to the tree shrew strains in China (green in Fig. 5). These data suggest that circulation and reassortment have occurred in East Asia at least.

The host species of the novel sewage isolates could not be determined in this study because of the wide range of host specificity of MRV infection. All three Osaka strains were isolated from children, implying that the THK0325 isolate also originated from humans, while the host of THK0617 is still unknown. Regardless, MRVs have the potential for zoonotic transmission.

Despite frequent isolation of MRV from human stool or wastewater samples^13,17,21^, this is the first report on the molecular characterisation of wastewater MRVs in Japan. Of note, the samples in this study were collected from the influent sewage of the WWTP, and it has been reported that MRVs are more sensitive to chlorine than enteroviruses during water disinfection^27^. RD cells utilised in polio surveillance are not permissive to MRV infection, whereas VeroE6 cells can be used to isolate a broad range of viruses such as severe acute respiratory syndrome coronavirus-2 (SARS-CoV-2) and MRV, although there are no reports on the successful isolation of SARS-CoV-2 from wastewater. It is possible that coronavirus disease-2019 environmental surveillance may increase the chance of unintentional isolation of MRV strains and accumulation of their genetic information.

## Materials and methods

### Sample collection, virus concentration, and isolation

Ten samples of influent wastewater (100 mL each) were collected weekly from a WWTP in the Tohoku area of Japan from March to June 2020. All samples were collected in sterile plastic bottles and immediately transported to the laboratory. During transportation, the samples were kept frozen at − 20 °C until analysis. Virus recovery from the collected wastewater was performed using polyethylene glycol (PEG) precipitation. After centrifugation at 3000 rpm (1840 × *g*) for 30 min to remove debris, PEG 8000 and NaCl were added to the supernatant (final concentrations were 10% and 1 M, respectively) and incubated at 4 °C overnight with gentle agitation. After centrifugation at 10,000 × *g* for 30 min, the PEG precipitant was dissolved in 500 µL of PBS (−). VeroE6 cells (ATCC CRL-1586), VeroE6/TMPRSS2 cells (JCRB1819), OUMS-36T-2 cells (JCRB1006.2), and HCT-8 cells (ATCC CCL-244) were used for virus isolation. The concentrated samples were inoculated into each cell culture and examined for CPE over a 14-day observation period. From the CPE positive culture, the supernatant was collected as the viral isolate.

### Transmission electron microscopy

Viral isolates from cell culture were concentrated using 7% PEG 8000. The pellet was dissolved in PBS (−) and the samples were further purified through 0.15 mL of Saphacryl S-400HR (GE HealthCare Life Sciences Co. Ltd) filled microspin columns and centrifuged at 800 × *g* for 2 min. The eluate was diluted tenfold with PBS (−). The sample was negatively stained with phosphotungstic acid and examined with a JEM-1400 Plus electron microscope (Japan Electron Optics Laboratory, Tokyo, Japan) at a magnification of 40,000×. The particle sizes were determined using JEM-1400 Plus software.

### Next generation sequencing

Viral RNA was extracted from the isolates using the High-Pure total RNA isolation kit (Roche), according to the manufacturer’s instructions. Sequence-independent amplification of viral RNA was performed as previously described^28^. The random amplicons were subjected to the Nextera XT DNA Library Prep Kit (Illumina), according to the manufacturer's instructions and sequenced using the MiSeq platform (Illumina). Raw sequencing reads were analysed using VirusTAP, a web-based integrated NGS analysis tool^22^. De novo assembled contigs were confirmed as all ten segments of MRV, although some of them covered a partial coding sequence. Reference-based mapping with the mammalian orthoreovirus genome was performed using CLC Genomics Workbench software with the default settings. The validity of the sequences from the reference-based mapping was confirmed with the initial de novo assembled contigs from the VirusTAP analysis. Complete or near-complete coding sequences of the ten segments of both isolates were obtained.

### Phylogenetic and homology analyses

The nucleotide sequences were analysed using BLASTn to determine the highest similarities to the sequences in the NCBI GenBank nucleotide database. MRV genomes of best hits from BLASTn searches and prototypes were obtained from the GenBank database. Multiple sequence alignments with the MUSCLE programme^29^ were performed using MEGA software ver. 10.1.7^30,31^. A phylogenetic tree was constructed by the maximum-likelihood method using the Tamura-Nei model^32^ and 1000 bootstrap replicates using MEGA. Homology analysis to calculate nucleotide and amino acid identities was performed using GENETYX-MAC software ver. 20 (GENETYX, Tokyo, Japan).

## Supplementary Information

Supplementary Figure 1.

## Data Availability

The GenBank/EMBL/DDBJ accession numbers for the sequences of the THK0325 and THK0617 isolates determined in this study are LC613209 to LC613218 and LC613219 to LC613228, respectively. Other datasets generated or analysed during the current study are available from the corresponding author upon reasonable request.
