# Supplementary material for: Intertypic reassortment of mammalian orthoreovirus identified in wastewater in Japan
Source: Sci Rep. 2021 Jun 15;11:12583. doi: 10.1038/s41598-021-92019-z (PMC8206364; doi:10.1038/s41598-021-92019-z)
Supplement: Supplementary file 1 — Supplementary Figure 1. [file 41598_2021_92019_MOESM1_ESM.docx]

Supplementary Figure 1

Title

Intertypic reassortment of mammalian orthoreovirus identified in wastewater in Japan

Authors

Kouichi Kitamura^1,†,*^, Hirotaka Takagi^2,†^, Tomoichiro Oka^1^, Michiyo Kataoka^3^, Yo Ueki^4^, Akie Sakagami^4^

Author Affiliations

^1^Department of Virology II, National Institute of Infectious Diseases, Tokyo 208-0011, Japan

^2^Management Department of Biosafety and Laboratory Animal, National Institute of Infectious Diseases, Tokyo 162-0052, Japan

^3^Department of Pathology, National Institute of Infectious Diseases, Tokyo 208-0011, Japan

^4^Miyagi Prefectural Institute of Public Health and Environment, Sendai 983-0836, Japan

^†^These authors contributed equally to this work

*To whom correspondence should be addressed:

Kouichi Kitamura

Department of Virology II, National Institute of Infectious Diseases, Murayama branch, 4-7-1 Gakuen, Musashi-murayama, Tokyo 208-0011, Japan

Tel: +81-42-561-0771

Fax: +81-42-561-4729

E-mail address: kkita@nih.go.jp

Supplementary Figure 1. Phylogenetic analysis based on the nucleotide sequences of novel MRV isolates. Phylogenetic trees for the S1 segment were constructed through the maximum-likelihood method and 1,000 bootstrap replicates using MEGA software. MRV-1 and MRV-2 sequences obtained from the NCBI Virus database (taxonomy: mammalian orthoreovirus). Each ID depicts the MRV genotype, host species, country, and accession number. Solid squares indicate the isolates in this study. The scale bar indicates genetic distances (nucleotide substitutions per site).
